# Random forest model in tax risk identification of real estate enterprise income tax

**DOI:** 10.1371/journal.pone.0300928

**Published:** 2024-03-26

**Authors:** Chunmei Xu, Yan Kong

**Affiliations:** 1 School of Business Administration, Guangxi Vocational & Technical Institute of Industry, Nanning, Guangxi, China; 2 Finance Department of Qufu Normal University, Qufu, Shandong, China; University of Cagliari: Universita degli Studi Di Cagliari, ITALY

## Abstract

The text describes improvements made to the random forest model to enhance its distinctiveness in addressing tax risks within the real estate industry, thereby tackling issues related to tax losses. Firstly, the paper introduces the potential application of the random forest model in identifying tax risks. Subsequently, the experimental analysis focuses on the selection of indicators for tax risk. Finally, the paper develops and utilizes actual taxpayer data to test a risk identification model, confirming its effectiveness. The experimental results indicate that the model’s output report includes basic taxpayer information, a summary of tax compliance risks, value-added tax refund situations, directions of suspicious items, and detailed information on common indicators. This paper comprehensively presents detailed taxpayer data, providing an intuitive understanding of tax-related risks. Additionally, the paper reveals the level of enterprise risk registration assessment, risk probability, risk value, and risk assessment ranking. Further analysis shows that enterprise risk points primarily exist in operating income, selling expenses, financial expenses, and total profit. Additionally, the results indicate significant differences between the model’s judgment values and declared values, especially in the high-risk probability of total operating income and profit. This implies a significant underreporting issue concerning corporate income tax for real estate enterprises. Therefore, this paper contributes to enhancing the identification of tax risks for real estate enterprises. Using the optimized random forest model makes it possible to accurately assess enterprises’ tax compliance risks and identify specific risk points.

## Introduction

Tax risk management plays a pivotal role in contemporary society, particularly within industries such as real estate enterprises, where tax compliance and risk identification assume paramount importance [1]. Given the intricate nature of business operations and the evolving landscape of tax-related policies, real estate enterprises encounter a myriad of potential tax risks. Consequently, the scientific and effective implementation of tax risk management holds immense significance for safeguarding enterprise interests, fostering tax equity, and ensuring stable economic development [2]. In response to these challenges, researchers continually endeavor to devise novel methods and models aimed at accurately assessing the tax-related risks associated with real estate enterprises and formulating targeted risk identification and management strategies. The optimized random forest algorithm, as an advanced data mining and analysis tool, has garnered widespread attention in the realm of tax risk management. Leveraging big data and self-learning mechanisms, this algorithm adeptly identifies risk points and furnishes quantitative risk assessment outcomes, thereby serving as a robust foundation for decision-making by tax authorities and taxpayers [3].

Strauss et al. (2020) scrutinized the value-added tax system, delving into its taxation principles, the concept of tax risk, tax risk management, and the risk management process and model as part of their investigation into tax risk identification. Categorizing tax risk based on causes, they identified policy risk stemming from policy gaps, tax collection risk arising from deficiencies in tax administration, and tax compliance risk originating from individual taxpayer motivations. The paper examined the manifestation and formation mechanism of tax risk following comprehensive tax reform [4]. Dwiantari and Artini (2021) employed case data analysis to elucidate the implementation of tax compliance management risk in the context of big data. Recognizing the underutilization of big data in tax compliance risk management due to factors such as a lack of awareness, ambiguous legal definitions, and the challenges of talent, data, and technology, the paper proposes recommendations to enhance the rationality and efficiency of tax compliance risk management, drawing insights from experiences in China and other countries [5]. In the domain of the random forest model, Santra (2022) developed and refined the random forest model and conducted empirical tests on three models utilizing 670 sets of samples from residential housing. The optimal model accurately predicted the market value of housing stock and calculated the housing tax base. The findings underscore the efficacy of enhancing the random forest model for precise predictions, suggesting its applicability in determining property tax bases. This paper contemplates the integration of the optimized random forest model and estimated average prices into a system for the collection and evaluation of property taxes [6].

This paper optimizes the random forest model through a systematic approach grounded in conventional research methodologies. The initial focus is directed towards elucidating the application of random forests in the context of tax risk identification. Subsequently, a thorough examination is conducted regarding the selection of tax risk indicators, culminating in the development of a corresponding risk identification model. The efficacy of the model is substantiated through simulations involving taxpayer data, thereby validating its applicability. The paper outlined in this paper not only charts a novel trajectory for refining the random forest model but also significantly enhances the discourse surrounding the identification of tax risks within enterprise contexts.

## Random forest model in the identification of income tax risk of real estate enterprises

### Real estate enterprise income tax risk index

**Fig 1:**
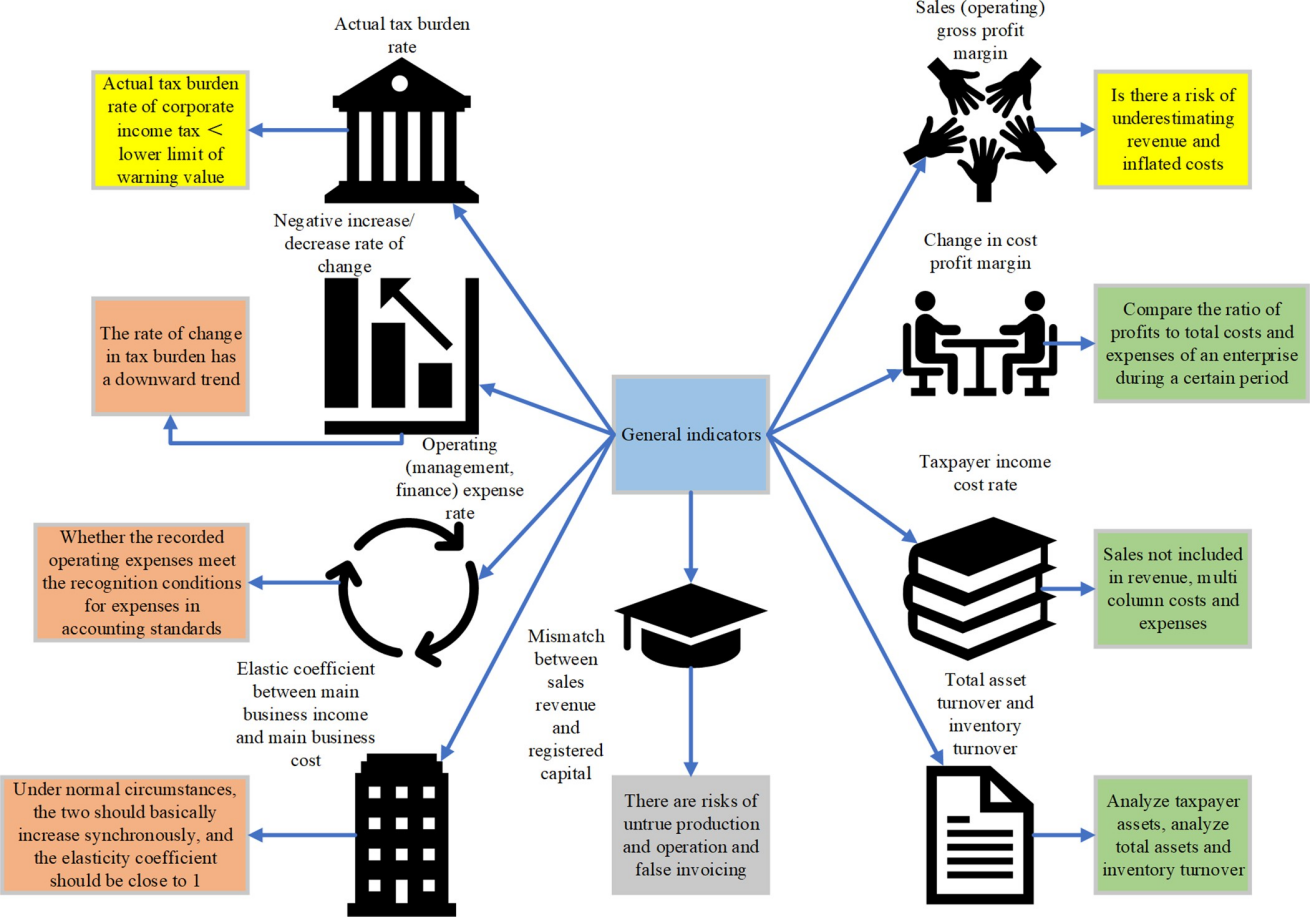
General indicators for the real estate industry.

The funding required for the provision of public amenities is acquired through the mandatory and gratuitous collection of taxes. However, the inherent nature of public goods often gives rise to instances of hitchhiking, resulting in potential conflicts between taxpayers and governmental entities, commonly referred to as tax risk. Tax risk is inherently a probabilistic event characterized by uncertainty [7]. Diminished tax compliance on the part of taxpayers may lead to increased income, establishing a scenario where neither the tax collector nor the taxpayer can entirely mitigate the risks associated with taxation [8–10]. Consequently, it is pragmatically imperative to explore tax-related concerns with the aim of augmenting tax compliance. The real estate industry, classified as an enterprise, encounters many of the conventional tax risks confronted by other industries. However, owing to its unique operational model and accounting procedures, the real estate sector also contends with distinctive tax risks. In the pursuit of scrutinizing corporate income tax risk within the real estate industry, this paper selectively examines a set of indicators encompassing both general and industry-specific considerations. Fig 1 illustrates the pertinent data pertaining to these general indicators.

**Fig 2:**
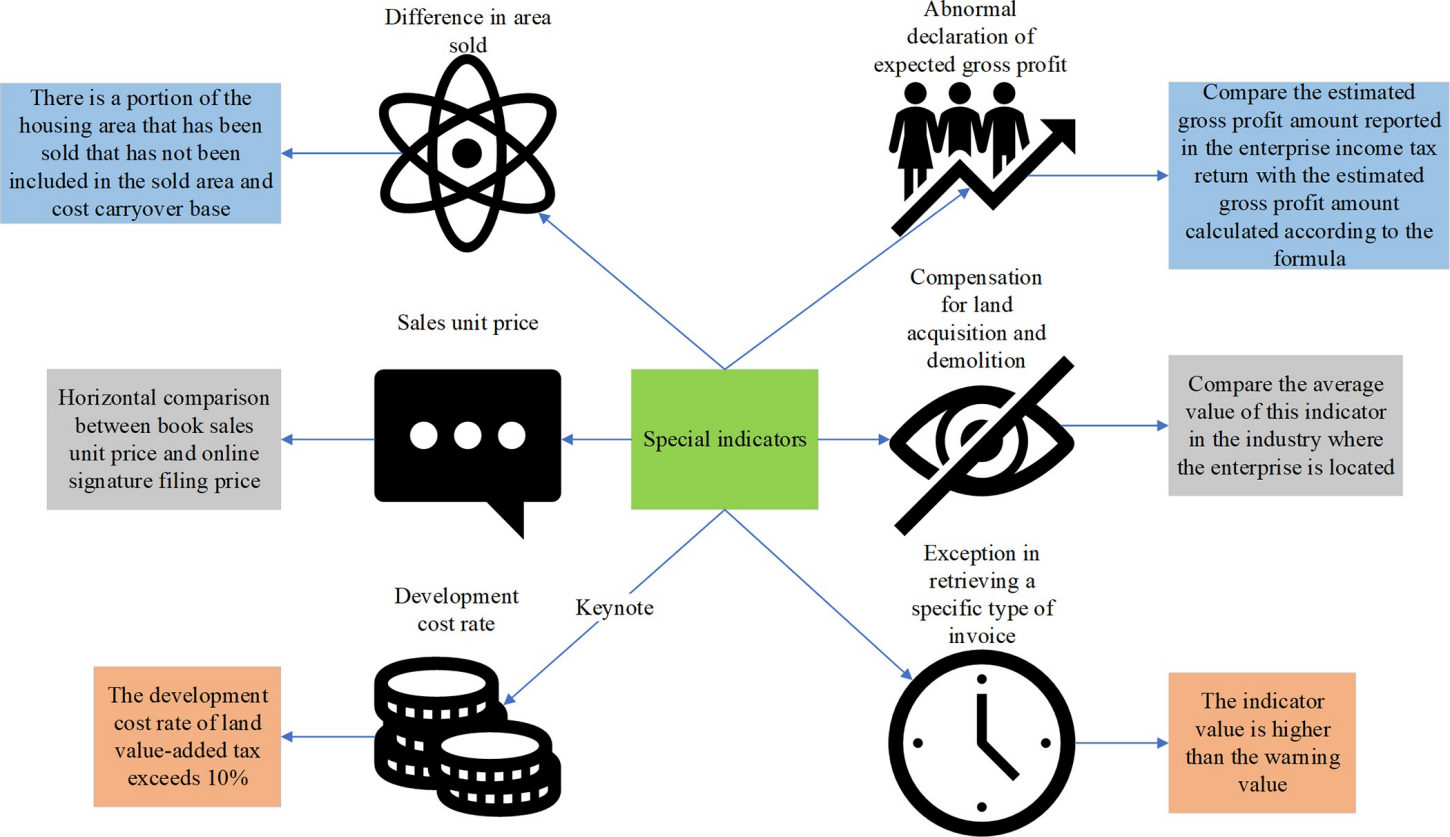
Special indicators for the real estate industry.

Fig 1 incorporates two supplementary elements alongside the fundamental indicators: the asset profit rate, total asset turnover rate, ratio analysis of sales profit rate, and the comparative assessment of income tax declaration income versus value-added tax declaration revenue. Their principal sources of concern encompass data pertaining to primary business income, primary business costs, raw materials, inventory items, and fixed assets [11]. Fig 2 delineates specific indicators relevant to the real estate industry.

Distinctive indicators play a crucial role in the financial scrutiny of real estate enterprises, with their balance sheets serving as the primary reservoir of data. The computation and evaluation of balance sheet data unveil critical details regarding the solvency, profitability, and return on investment of real estate firms. Among these, certain issues necessitate focused attention on specialized indicators, such as the development expense rate and anomalous reporting of expected gross profit. Notably, development costs and land value-added tax assume prominence due to their direct impact on the operational costs and income tax liabilities of real estate enterprises. Consequently, for a more precise assessment of the financial standing and operational risks of real estate firms, particular emphasis should be placed on these concerns within the framework of specialized indicator analysis. It is imperative to conduct reasoned analysis and judgment in alignment with the prevailing circumstances. The utilization of information gain proves instrumental in enhancing the purity of the dataset subsequent to feature partitioning, thereby aiding in the identification of optimal partitioning features.


G(D,A)=E(D)−∑(D,v)*E(D,v) (1)

In Eq (1), *E*(*D*) denotes the entropy associated with the dataset; (*D,v*) signifies the sub-dataset resulting from division by a specific value of the feature; *A* denotes the feature under consideration; and *G* represents the gain process. The Out of Bag (OOB) error emerges as a technique employed to assess the efficacy of the random forest model. This methodology leverages OOB sample data, which remains unutilized during the training phase, to gauge the model’s predictive accuracy.


P=1−(U/C) (2)

In Eq (2), *P* denotes the OOB error, where *U* represents the count of correctly predicted OOB samples, and C signifies the total number of OOB samples. The computation of the OOB error yields an assessment metric independent of an additional validation set, providing a measure of the random forest model’s predictive performance on unseen samples. A diminished OOB error typically signifies enhanced model generalization and prediction accuracy. This facilitates the evaluation and comparison of diverse models, aiding in the optimal selection of model parameters.

### Random forest model in tax risk identification

**Fig 3:**
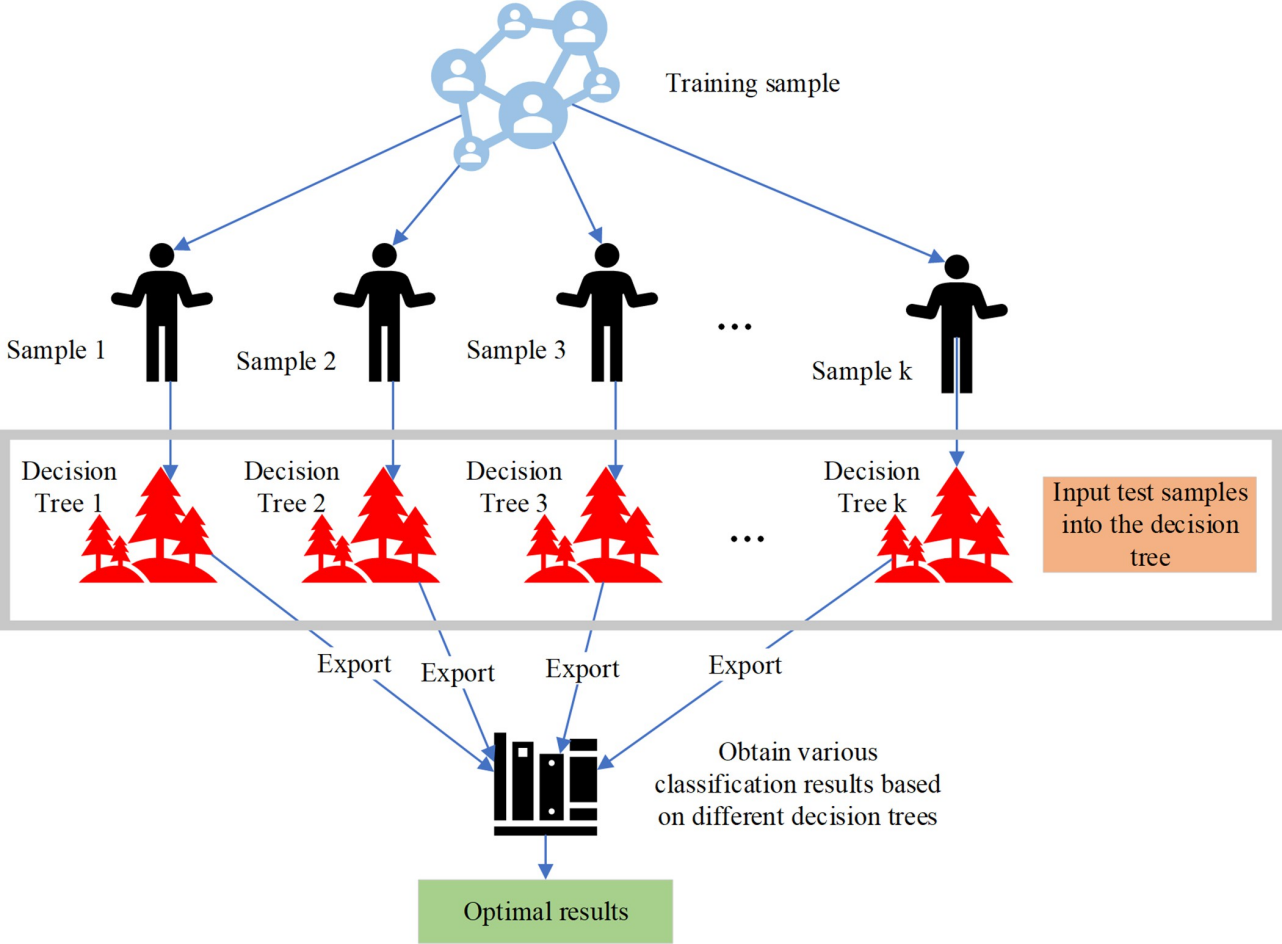
Random forest model process.

A consolidated approach, specifically a Bagging type known as the random forest, amalgamates multiple classifiers, each individually ineffective. The cumulative results of the model exhibit heightened accuracy and universality through a process of voting or averaging during generalization performance [12]. The random forest employs a collection of decision trees, selected at random, wherein each tree is trained on distinct samples, yielding decision trees with unique features. This method amplifies model categorization by accentuating distinctions among individual models [13]. In order to assess and fortify the model’s resistance to transformations or enhance its robustness, the Random Forest adopts the pocket-out-of-bag approach [14]. Given that each variable can train a substantial number of samples, the random forest’s multi-decision tree structure ensures the utilization of multiple variables in tax assessment. This enables the random forest to amalgamate unrelated variables into a concise set, maximizing the utilization of data compiled by tax authorities [15]. Through collective voting, the model can more accurately ascertain the potential risk of taxpayers by training on numerous historical samples [16]. The flowchart delineated in Fig 3 illustrates the procedural sequence.

Fig 3 illustrates that the random forest model, through the proficient utilization of extensive taxpayer data and a robust decision tree integration method, can expeditiously and precisely discern potential tax risks in the process of tax risk identification. The model initiates training on samples using historical data of real estate enterprise income tax, comprising diverse taxpayer characteristics and the presence of tax risks. Subsequently, each sub-sample is employed to train an individual decision tree, ensuring that each tree learns based on distinct subsets of data. Each tree independently learns how to classify data from its sample, potentially resulting in variations in structure and acquired knowledge. Once all decision trees are trained, they are utilized to classify new test samples. Each decision tree provides its classification, and these results are aggregated. By voting or averaging these results, the final prediction is obtained. This process aids in reducing overfitting and enhancing the model’s generalization capacity to unseen data. By amalgamating predictions from all decision trees, the random forest model furnishes the ultimate determination of tax risk for new samples. This result is considered "optimal" as it consolidates knowledge from multiple models, often yielding more accurate and robust predictions compared to individual decision trees.

This model process enhances the efficiency and accuracy of tax risk management, providing robust decision support for tax departments and taxpayers, thereby fostering tax compliance and ensuring stable economic development. Currently, the predominant assessment indices for classification models include accuracy, precision, recall, and F1-Measure [17]. The identification of income tax risk in real estate firms integrates theories of tax risk management, optimal taxation, and information asymmetry [18]. Tax risk management theory posits that the enhancement of tax risk management involves defining objectives, establishing relevant standards and systems, analyzing and identifying tax risks, categorizing risk levels, and effectively controlling and eliminating tax risks. Furthermore, it emphasizes refining and perfecting the tax risk management process through post-event performance evaluation [19]. Tax authorities frequently utilize this theory to allocate resources judiciously by discovering, ranking, and managing risks through monitoring, assessment, and other processes. The development of this hypothesis is a result of the organic growth of contemporary information technology and tax management techniques [20].

The goal of optimal taxation theory is to comprehend how to balance, select, and coordinate relationships between different tax principles, designing the tax system scientifically to make taxes advantageous while avoiding negative effects [21]. The fairness principle and efficiency principle are central tenets of optimal taxes. Equal tax design aligns with the fairness principle, and efficiency is achieved by minimizing the cost of collection and management to enhance administrative efficiency or mitigating the negative incentive effects of taxes on the economy to improve economic efficiency [22]. The optimal taxation theory asserts that the government must bear the excessive burden of taxation as the price for levying taxes. Hence, it is imperative to devise a taxation strategy capable of collecting necessary revenue while minimizing this excessive burden [23]. According to the information asymmetry theory, different subject categories possess varying levels of knowledge, with those having more information generally holding an advantageous position, while those with less information are at a disadvantage [24]. In economic markets, knowledge asymmetry is prevalent, where tax authorities, apart from knowing more about their products than customers, also have limited insight into company information compared to businesses [25]. Information asymmetry results in varied risks and rewards for different subjects, influencing market fairness and economic operation significantly through government regulation of economic activity [26].

### Design of real estate enterprise income tax risk identification model based on random forest model

Enterprise income tax, a vital tool for tax adjustment and distribution, serves as a direct tax that reflects the state’s involvement in the allocation of social tax funds [27]. Distinguished from other taxes, the management of enterprise income tax presents unique challenges. Net income reported monthly and quarterly in advance, serves as the basis for calculating taxable income, settled at the year’s end. Characterized by a high tax burden that is challenging to shift, enterprise income tax exhibits increased risk points due to the flexible tax burden and intricate, evolving preferential tax policies [28]. Managing enterprise income tax risk faces challenges such as poor-quality basic data due to erroneous taxpayer reporting, limited sophistication and comprehensiveness in tools for analyzing enterprise income tax data, minimal use of indicators, and a heavy reliance on fabricated experience. Thus, the complexity and mutability of enterprise income tax pose persistent challenges in practical risk management [29]. The real estate industry encompasses both physical and financial aspects, featuring a complex internal financial processing system. Coupled with extended development cycles, improper financial information maintenance, business-level disparities, and job turnover among financial personnel can easily lead to data inaccuracies, impacting tax authorities’ inspection efforts [30]. Consequently, timely and efficient detection of corporate income tax risk in the real estate market is paramount [31].

**Fig 4:**
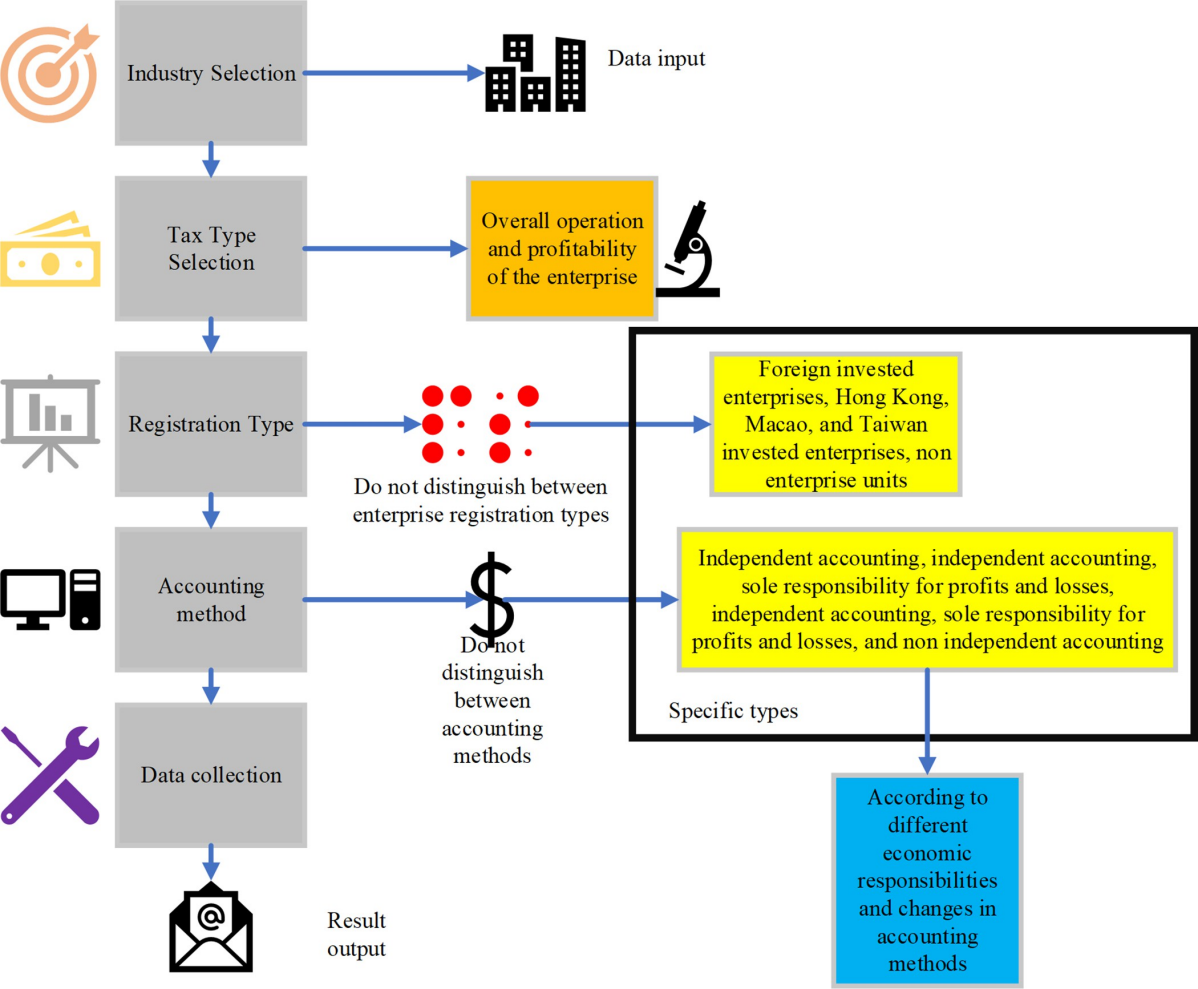
Optimized random forest model process.

This paper selects numerous risk indicators from the perspectives of assets, revenue, cost, and expense. These indicators include examinations of ratios for total asset turnover, income cost rate, capital profit rate, and sales profit rate; elasticity coefficient between primary business revenue and primary costs; management fee rates; gross profit margin of sales (for Business); income tax real tax burden rate; adjustments in cost, expense, and profit rates; and a comparison of income from VAT declaration with income from enterprise income tax declaration. These chosen indicators further quantify the outcomes of risk identification, illustrating the locations of tax risk sites. In order to initiate model training, the risk model’s definition is established by screening risk indicators based on corresponding tax data dimensions, such as provincial tax authorities, competent tax authorities, municipal tax authorities, indicators, data period, tax type, industry, tax registration type, accounting method, taxpayer scale, and data set. Fig 4 delineates the risk identification model based on the random forest model employed in this paper.

The model’s test set predictions depicted in Fig 4 provide multi-dimensional numerical feedback, revealing the taxpayer’s risk situation. Traditional tax risk prevention and control systems typically employ risk intervention measures during and after events, yielding effective feedback only after the risk has materialized, representing a more passive risk management approach. Preceding the introduction of artificial intelligence (AI) and advanced algorithms, tax authorities attempted to use alternative technical means for early sensitive predictions to proactively address potential tax risks. However, the efficacy of these predictions was limited, primarily enhancing risk identification abilities in data quality but falling short in fully realizing the role of pre-prevention and control of tax risks. The development of AI technology and the application of advanced algorithms, such as the random forest in model testing, have yielded positive results. Through extensive machine learning training, the computer autonomously generates comprehensive risk prediction results without human intervention, encompassing model construction, algorithm time complexity, and space complexity selections. These projections are machine-generated predictions based on taxpayer behavior. For instance, for a taxpayer exhibiting fraud risk, the model provides the probability of future fraud and the corresponding fraudulent amount in the test set prediction results.

Within the tax risk prevention and control system, the model test results are tailored to tax business, defining data analysis objectives and analysis logic. It integrates risks occurring before, during, and after events to formulate a comprehensive and unified tax risk analysis report. This facilitates a detailed display of taxpayer data through specific content output by the model’s report, including the taxpayer’s basic information, tax compliance risk summary, value-added tax return, direction of doubtful subjects, and detailed information on common indicators. This approach enhances the intuitive understanding of tax-related risks.

In summary, the advancement of AI technology and the utilization of advanced algorithms, such as the random forest in model testing, have demonstrated positive effects on tax risk management. This method surpasses traditional early warning approaches, which rely solely on rigid indices compiled by the paradigm, enabling more effective pre-prevention and control of tax risks. Through this technical means, tax risks can be identified and managed at an earlier stage, contributing to improved tax compliance levels and furthering tax fairness and sustainable economic development.

## The identification results of income tax risk of real estate enterprises under the random forest model

### Basic information on taxpayers and risk summary of tax compliance in real estate enterprises

Taxpayers are categorized into 1–9 different risk levels by implementing a risk ranking experiment. The highest risk level is denoted as 9, with the subsequent levels decreasing sequentially. In the model testing phase, the experiment primarily focuses on taxpayers with risk levels 9, 8, and 7, encompassing over 100 households in the real estate industry. Following the principles of risk management and business rules, both summary and individual household reports are generated, and the data results of the real estate industry model analysis are analyzed and applied. Due to confidentiality considerations, certain financial data and identification results cannot be disclosed in this paper. However, this limitation does not compromise the presentation of identification results. Table 1 provides an overview of the basic information of taxpayers.

**Table 1:** Basic information of taxpayers.

Information project	Essential information
Enterprise Name	*****Limited Company
Date of Incorporation	****-*-*
Listed company or not	Deny
Registration Type	Private limited liability company
Number of people engaged	****
Total assets	*,***,***
Accounting Standards for Business Enterprises	General enterprises
Unified social credit code	**************
Business status	********
Legal representative	***
Corporate Phone	**********

**Fig 5:**
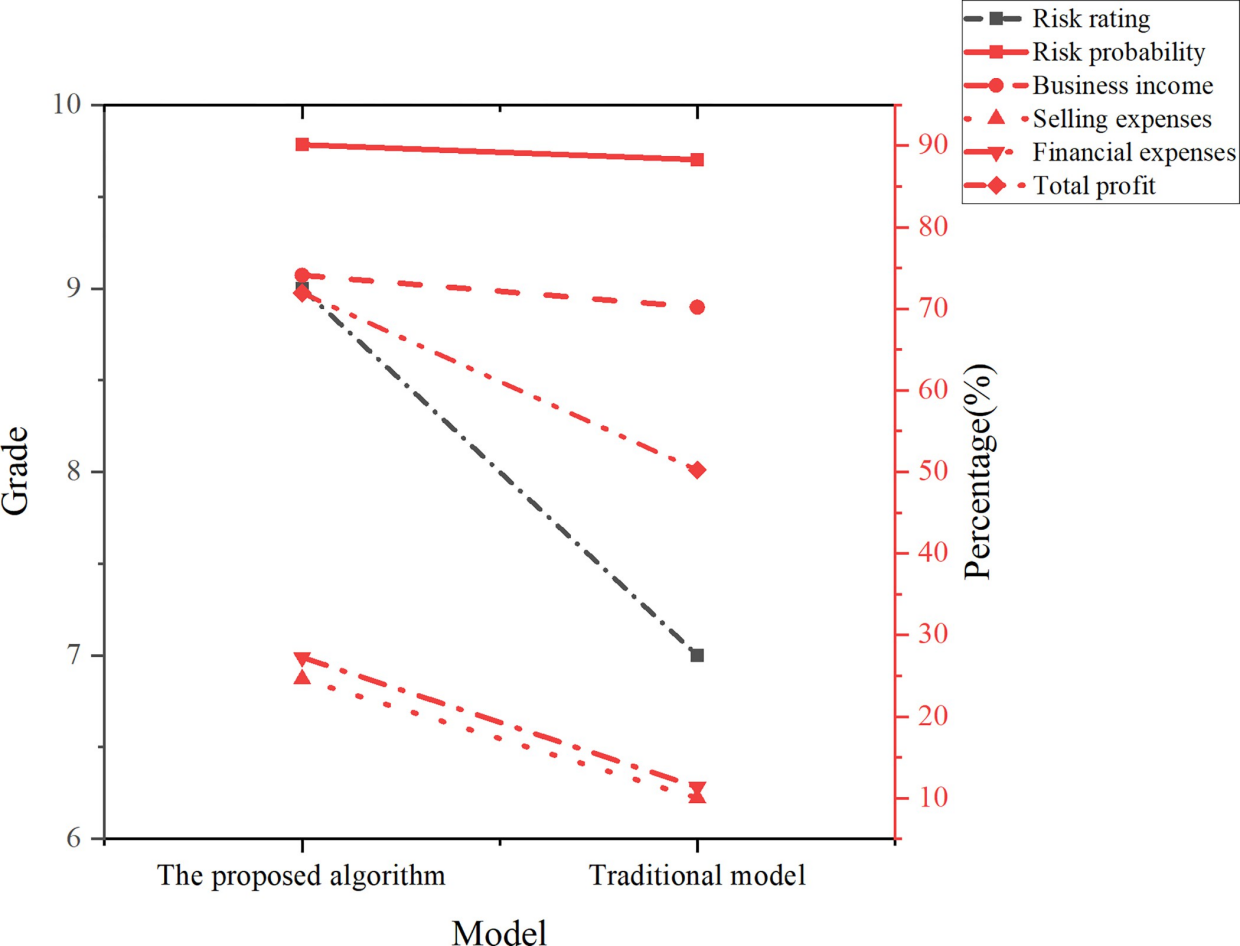
Summary of tax compliance risks.

Table 1 showcases the comprehensive basic information derived from the model. Simultaneously, it is important to note that the real estate enterprise operates as a private limited liability company. As a general taxpayer, its financial processing is relatively formal and holds reference value. In consideration of privacy protection, certain content undergoes private processing, although this processing does not pertain to the application and analysis of the data. The summarized analysis results of tax compliance risk are illustrated in Fig 5.

According to the findings in Fig 5, the enterprise’s risk level is determined to be 9, with a risk probability of 90.12%, ranking highest among all risk assessments. The analysis indicates that the primary risk points lie in financial expenses, business income, selling expenses, and total profit. Specifically, the risk probabilities for business income, selling expenses, financial expenses, and total profit are 74.11%, 24.59%, 27.25%, and 71.94%, respectively. Notably, there is a significant disparity between the declared value and the model’s judgment value. The elevated risk probabilities for business income and total profit suggest a notable issue of underpayment of enterprise income tax within the real estate enterprise. Furthermore, the risk direction highlights the undercounting of business income and overcounting of sales expenses and financial expenses.

In summary, the application of the random forest model proves effective in identifying income tax risks for real estate enterprises. The model results underscore a high-risk probability for the enterprise, particularly concerning business income and total profit. These insights carry significant implications for the enterprise’s financial management and tax declaration processes. It is imperative to enhance the verification of business income and implement strict controls over the recording and accounting of selling expenses and financial expenses. Concurrently, it is recommended that the enterprise fortify internal control measures to ensure accurate income tax declaration and payment, thereby mitigating potential legal and financial risks.

### The tax return, the direction of doubtful subjects, and the details of common indicators

**Fig 6:**
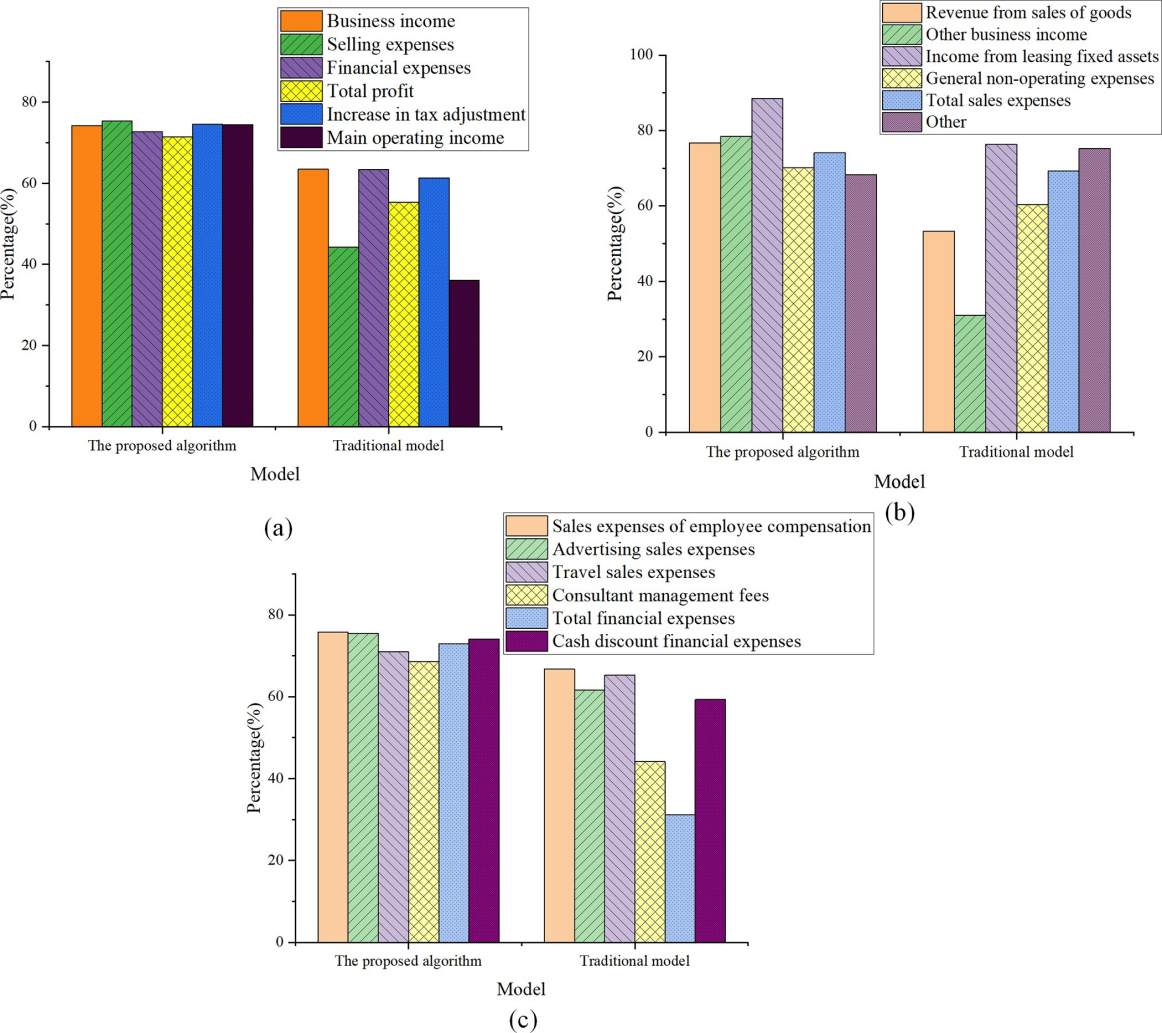
Tax declaration and doubtful subject pointing risk probability (a) General enterprise income and doubtful subject direction (b) General enterprise costs and doubtful accounts (c) Enterprise period expenses and doubtful items.

Through the analysis of tax returns and questionable subjects, the model’s predicted value is compared with the enterprise income tax reported by taxpayers, resulting in the determination of the risk probability. The outcome is illustrated in Fig 6.

Fig 6 shows that in the analysis of general corporate income and questionable items, the risk level of business income discrepancies is the highest, reaching 78.21%. Issues that may be involved include failure to timely recognize revenue from completed projects, failure to recognize various fee incomes collected, non-compliance with prescribed sales processing, failure to confirm bank mortgage loans and pre-sale income, potential failure to recognize detailed decoration income, whether lease income has been declared, whether income from relocated housing has been accounted for and whether government incentives and refunds have been recorded. Specific risk points need to be determined through on-site inspection of the real estate enterprise’s accounting information. In the analysis of corporate cost expenditures and questionable items, the risk probability of non-operating expenses reaches 70.2%. Non-operating expense discrepancies are significant, and potential issues may include whether asset restructuring has been conducted during the tax period, the authenticity and effectiveness of asset inventory, whether deductions for donations comply with tax law policies, the existence of extraordinary losses due to force majeure factors such as natural disasters, whether impairment provisions comply with accounting standards, and whether the disposal of fixed assets and intangible assets adheres to disposal processes. Similarly, on-site verification of the real estate enterprise’s accounting information is required to determine specific risk points. In the analysis of corporate expenses and questionable items, the risk probabilities for selling expenses, management expenses, and financial expenses are 74.1%, 68.65%, and 72.91%, respectively. Risks may include model house decoration costs being directly included in selling expenses rather than development costs; management expenses not being included in operating expenses and sponsorship expenses; loan interest expenses and amortization deductions not complying with policy regulations and accounting standards; whether employee welfare expenses have undergone tax adjustments, and whether leasing expenses for the sales department have been amortized during the lease period. To address these expenses prone to tax issues, it is also necessary to determine the enterprise’s operational status.

In summary, the analytical outcomes indicate a substantial risk probability for the real estate enterprise in categories such as business income, non-business expenses, selling expenses, management expenses, and financial expenses. To pinpoint specific risk areas, a comprehensive examination of the enterprise’s accounting information is imperative. These analytical findings serve as valuable tools for tax authorities in categorizing and overseeing taxpayers with varying risk levels. Furthermore, they offer guidance and recommendations to enterprises concerning tax declaration and accounting practices. The detailed analysis results of commonly utilized indicators are presented in Table 2.

**Table 2:** Common indicator details.

Indicator Name	Risk points
Abnormal total asset turnover rate	Risks of underestimating operating income, falsely increasing costs, and underreporting taxable income
Abnormal revenue cost rate	The risk of falsely increasing operating costs and underestimating operating revenue
Analysis of the matching of the asset profit rate, total asset turnover rate, and sales profit rate	There is a problem with concealing sales revenue.
Abnormal elasticity coefficient between main business income and main business cost	Multi-column cost expense to expand the scope of pre-tax deduction
Abnormal management expense rate	Overcounting management expenses
Abnormal sales gross profit margin	Risk of inflated costs
Abnormal actual tax burden rate of corporate income tax	Actual tax underpayment and income tax payable
Abnormal comparison between VAT declaration income and corporate income declaration income	Risk of concealing income
Cost profit margin becomes abnormal.	The ratio of a company’s profit to cost and total expenses over a certain period of time and finding abnormal data on changes

The optimization model utilized in the analysis presented in this paper is delineated in Table 2, aiming to accurately depict the enterprise’s potential risk factors and issues. In the context of a real estate development firm, the taxpayer, as revealed by the comprehensive model output, has failed to fulfill specific tax obligations mandated by relevant laws. The information return model, leveraging the random forest algorithm, adeptly identifies pertinent indicators. The prowess of AI-based risk identification in business applications is manifest in the model’s capacity to forecast the taxpayer’s risk probability. These predictive outcomes not only aid tax authorities in discerning the taxpayer’s current risk but also proactively comprehend the prognosis of potential future risks. This intelligent analytical approach significantly enhances the efficacy of risk screening conducted by tax inspectors and other departments for taxpayers. The outcomes of risk identification serve as substantiation and guidance in addressing the specific hazards confronting taxpayers.

### Discussion on the identification results of income tax risk of real estate enterprises

Based on the summarized analysis outcomes of tax compliance risk, noteworthy disparities emerge between the declared values of enterprises and the model’s evaluative values. Notably, there exists a heightened risk probability, particularly in the realms of business income and total profit. This implies a conspicuous issue of corporate income tax underpayment within the real estate enterprise. The risk orientation points towards the underestimation of business income and the overestimation of selling expenses and financial expenses. The optimization model employed in this paper adeptly delineates enterprises’ risk focal points and potential issues through the scrutiny of common indicators. Additionally, the comprehensive model analysis divulges that, in the course of operating a real estate development business, the taxpayer faces the risk of non-compliance with specific tax regulations. Simultaneously, the application of the random forest algorithm in the information return model allows for the incorporation of multiple risk indicators.

In contrast to traditional tax risk identification models, the random forest algorithm, grounded in data, utilizes program algorithms and computer technology, continuously refining itself through the learning process. It exhibits the capacity to discern and analyze the tax landscape of real estate enterprises within a defined scope, sorting entities based on risk identification outcomes to pinpoint those with higher tax-related risks. Confronted with extensive data, the algorithm crafts a recognition model through autonomous learning, yielding quantifiable risk values and probabilities. This transformation mitigates the issues of subjective human experience and uncertainty inherent in traditional models, yielding visualized and digitized outcomes. Moreover, the continuous self-learning feature of the random forest algorithm ensures a perpetual enhancement in the accuracy and quality of the risk identification model. Effectively identifying high-risk tax-paying enterprises and tax-paying risk points, the random forest algorithm empowers tax authorities to efficiently and accurately categorize and manage taxpayers with varying risk levels. Comparative to Wei et al.’s (2022) tax risk identification model, the model proposed in this paper excels in stability and accuracy [32]. Furthermore, when juxtaposed with Zhao’s (2022) tax risk identification model, the optimized model exhibits a heightened ability to capture key risk factors [33].

## Conclusion

The continuous evolution of science and technology has substantially enhanced the capabilities of the random forest model. Consequently, this paper undertakes an optimization of the random forest model to augment the precision of tax risk identification and enhance its applicability in this domain. Commencing with an exploration of tax risk indicators, the subsequent sections involve the formulation of a risk identification model. The efficacy and credibility of the model are rigorously tested through simulation experiments employing real taxpayer data, addressing the pertinent issue of tax loss within the real estate industry. The experimental outcomes underscore the utility of the random forest model in identifying income tax risks specific to real estate enterprises, providing a comprehensive risk report. This model facilitates the assessment of risk registration level, probability, value, and ranking for enterprises. Analysis of pertinent data for a given real estate enterprise reveals a high rating in risk assessment, securing the foremost position in the risk ranking. The identified risk points primarily pertain to business income, selling expenses, financial expenses, and total profits, with respective risk probabilities of 74.11%, 24.59%, 27.25%, and 71.94%. The substantial variance between the declared value and the model’s judgment, particularly the elevated risk probability associated with business income and total profit, suggests potential issues of underreported corporate income tax. Specifically, the risk points towards the underestimation of business income and overestimation of selling expenses and financial expenses, thereby validating the rationality and reliability of the proposed model. Distinguishing itself from traditional classification models, the random forest model boasts superior accuracy, rapid learning capabilities, and adept handling of high-dimensional data. The intricate nature of real estate industry data, characterized by strong industrial correlations, protracted project cycles, extensive temporal spans, substantial capital volumes, and intricate fundraising channels, underscores the random forest model’s effectiveness in identifying real estate enterprises, particularly those with missing data.

However, the paper is not without its challenges. On one front, the limited availability of third-party data prompts consideration of how to comprehensively gather data and establish a robust database. On another front, the existing deficiency in risk indicator features necessitates a concerted effort to refine the tax risk indicator system. Subsequent paper endeavors will encompass the incorporation of specialized indicators to address these issues comprehensively.

## Supporting information

S1 Data (ZIP)
