# Supplementary material for: Random forest model in tax risk identification of real estate enterprise income tax
Source: PLoS One. 2024 Mar 26;19(3):e0300928. doi: 10.1371/journal.pone.0300928 (PMC10965060; doi:10.1371/journal.pone.0300928)
Supplement: S1 Data — (ZIP) [file pone.0300928.s001.zip › ╩2╛▌░n/Code description.docx]

1. First, we prepare the necessary tools and algorithms by importing relevant modules from `pandas` and `sklearn`.

2. Then, we define a function called `feature_selection` for handling the feature selection process. Within the function, data preprocessing and cleaning operations can be performed. It selects the desired feature columns and returns the selected features and target column.

3. Next, we import the data file using the `pd.read_csv()` function and store the data in the `data` variable.

4. Call the `feature_selection` function to perform feature selection and save the returned feature columns in the `features` variable and the target column in the `target` variable.

5. Further preprocess the features, such as data cleaning and filling missing values (specific code examples for this part are not given).

6. Create a `RandomForestClassifier` object `rf_model` and set model parameters, such as the number of decision trees and the random seed.

7. Split the dataset using the `train_test_split` function into training and testing sets, and store the split data in the corresponding variables (e.g., `X_train`, `X_test`, `y_train`, `y_test`).

8. Train the random forest model using the training dataset by calling the `fit` method and passing the feature data `features` and target data `target`.

9. Perform hyperparameter tuning on the model using `GridSearchCV`. This involves trying different parameter combinations by specifying the parameter grid `param_grid` and evaluation metric `scoring`. Create a `GridSearchCV` object `grid_search`, then call the `fit` method with the feature data `features` and target data `target` to perform training and evaluation, searching for the best parameter combination during the cross-validation process.

10. Retrieve the best parameter combination using `grid_search.best_estimator_` and `grid_search.best_params_`, and rebuild the random forest model `best_rf_model` with these parameters.

11. Use the testing dataset to make predictions with the model by calling the `predict` method and passing the test set feature data `X_test`.

12. Calculate the accuracy of the model's predictions by calling the `accuracy_score` function and passing the predicted results `y_pred` and the actual labels `y_test`.

13. Print the accuracy evaluation result of the model, displaying the accuracy value.

14. Optionally, print the classification report by calling the `classification_report` function and passing the true labels `y_test` and predicted results `y_pred` for evaluation.

15. Optionally, print the confusion matrix by calling the `confusion_matrix` function and passing the true labels `y_test` and predicted results `y_pred` for evaluation.
