# Supplementary figures and images for: Random forest model in tax risk identification of real estate enterprise income tax
Source: PLoS One. 2024 Mar 26;19(3):e0300928. doi: 10.1371/journal.pone.0300928 (PMC10965060; doi:10.1371/journal.pone.0300928)

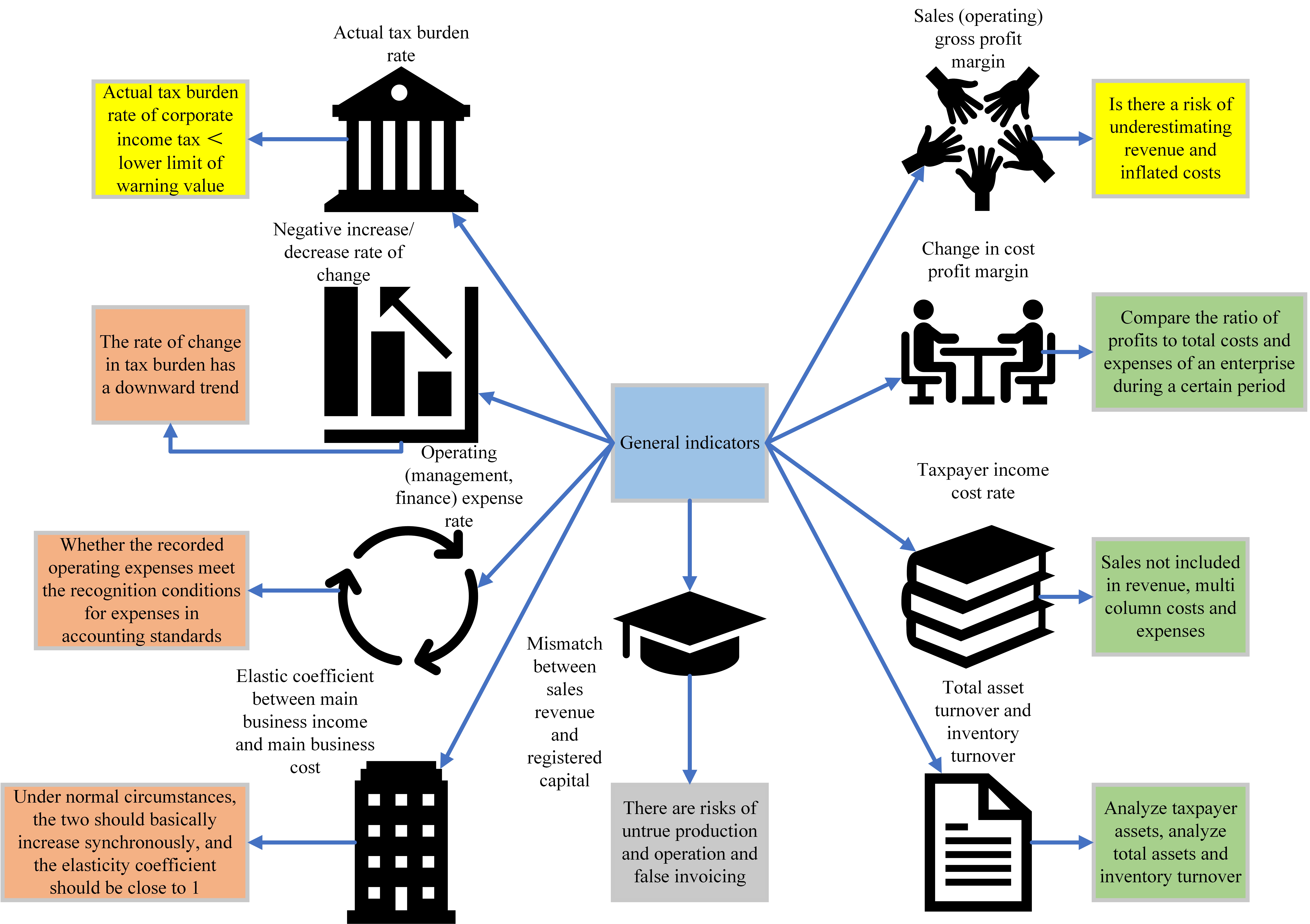

Supplement: S1 Data — (ZIP) [file pone.0300928.s001.zip › ╩2╛▌░n/Figure1.jpg]

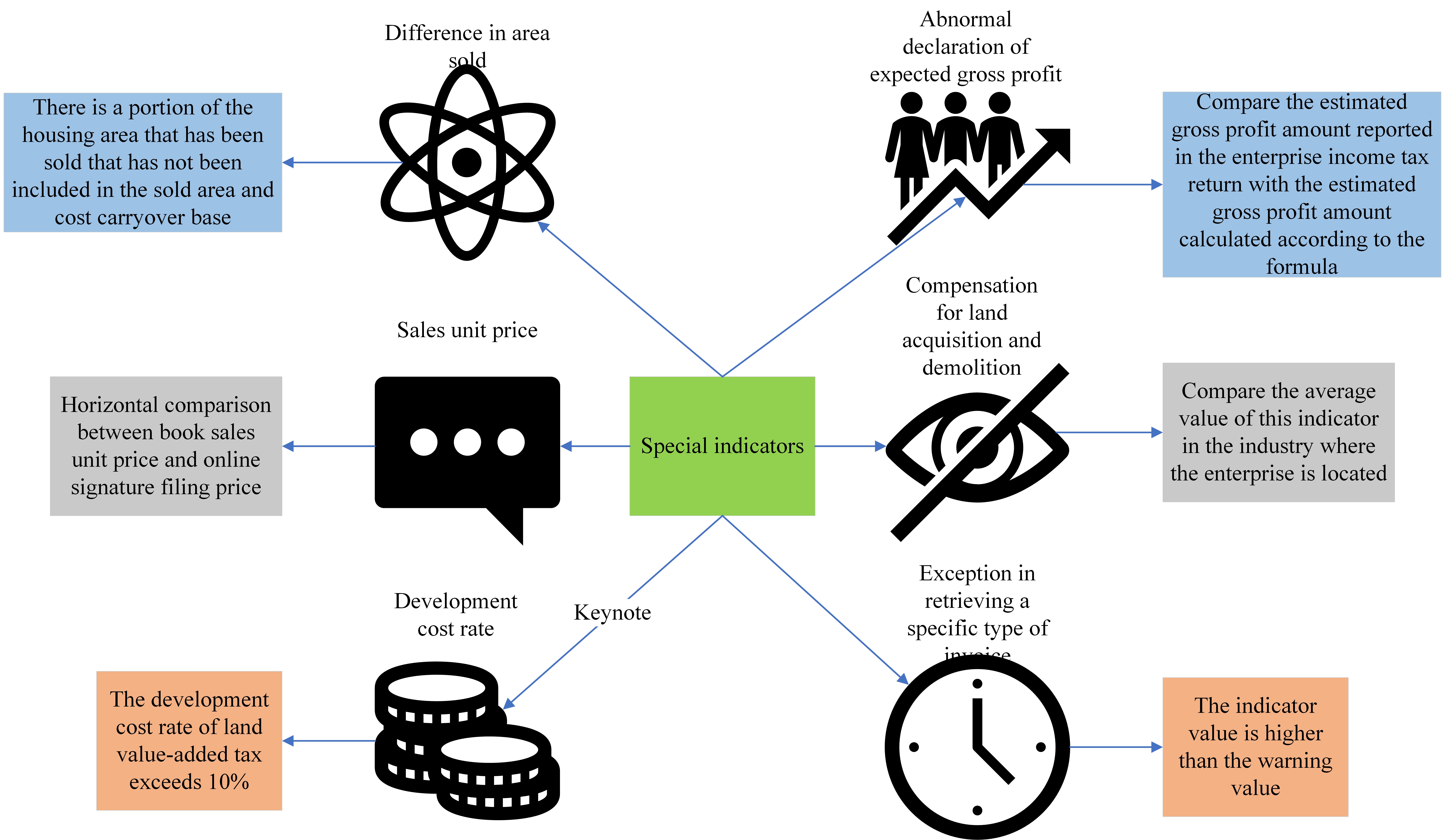

Supplement: S1 Data — (ZIP) [file pone.0300928.s001.zip › ╩2╛▌░n/Figure2.jpg]

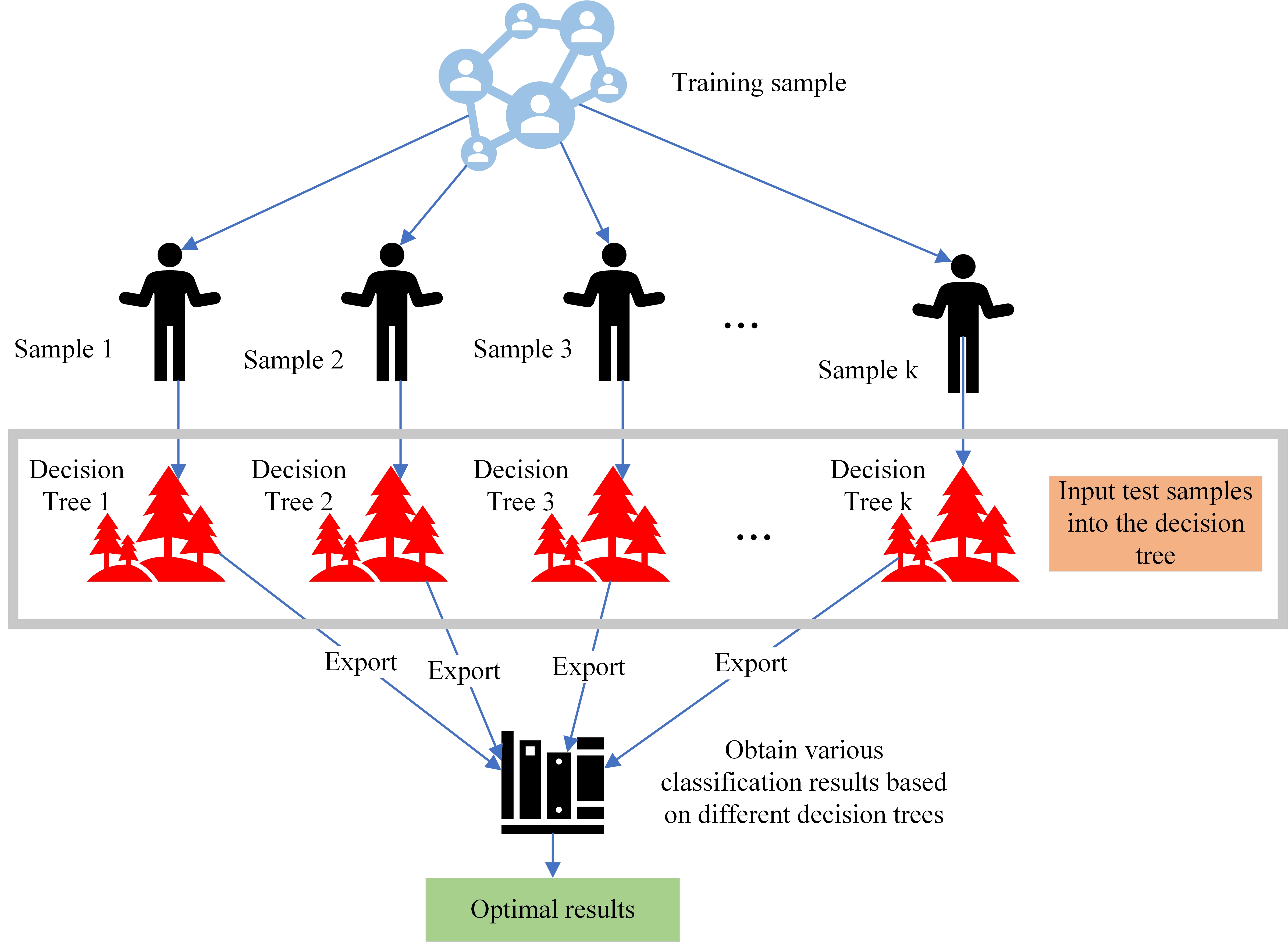

Supplement: S1 Data — (ZIP) [file pone.0300928.s001.zip › ╩2╛▌░n/Figure3.jpg]

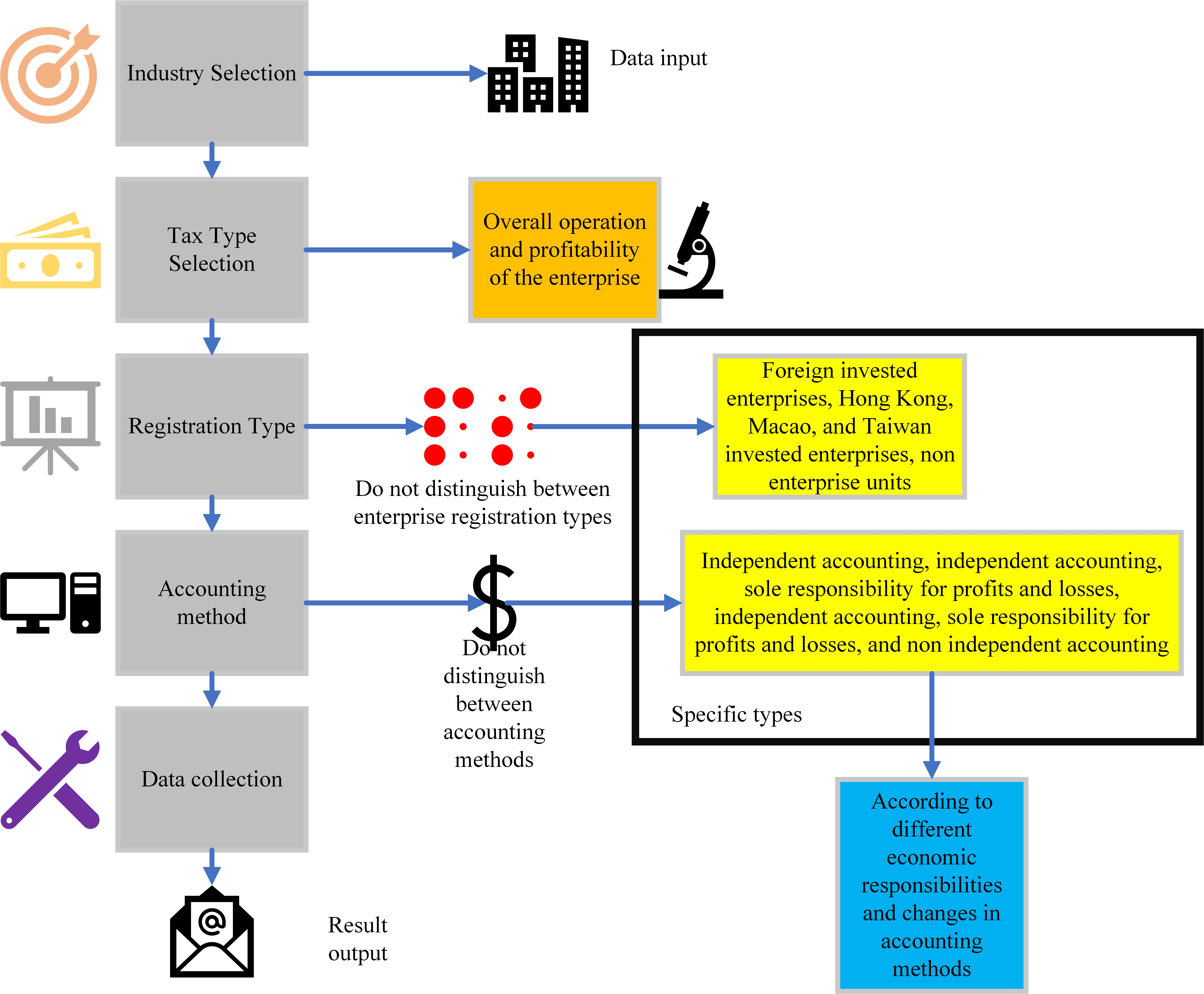

Supplement: S1 Data — (ZIP) [file pone.0300928.s001.zip › ╩2╛▌░n/Figure4.jpg]

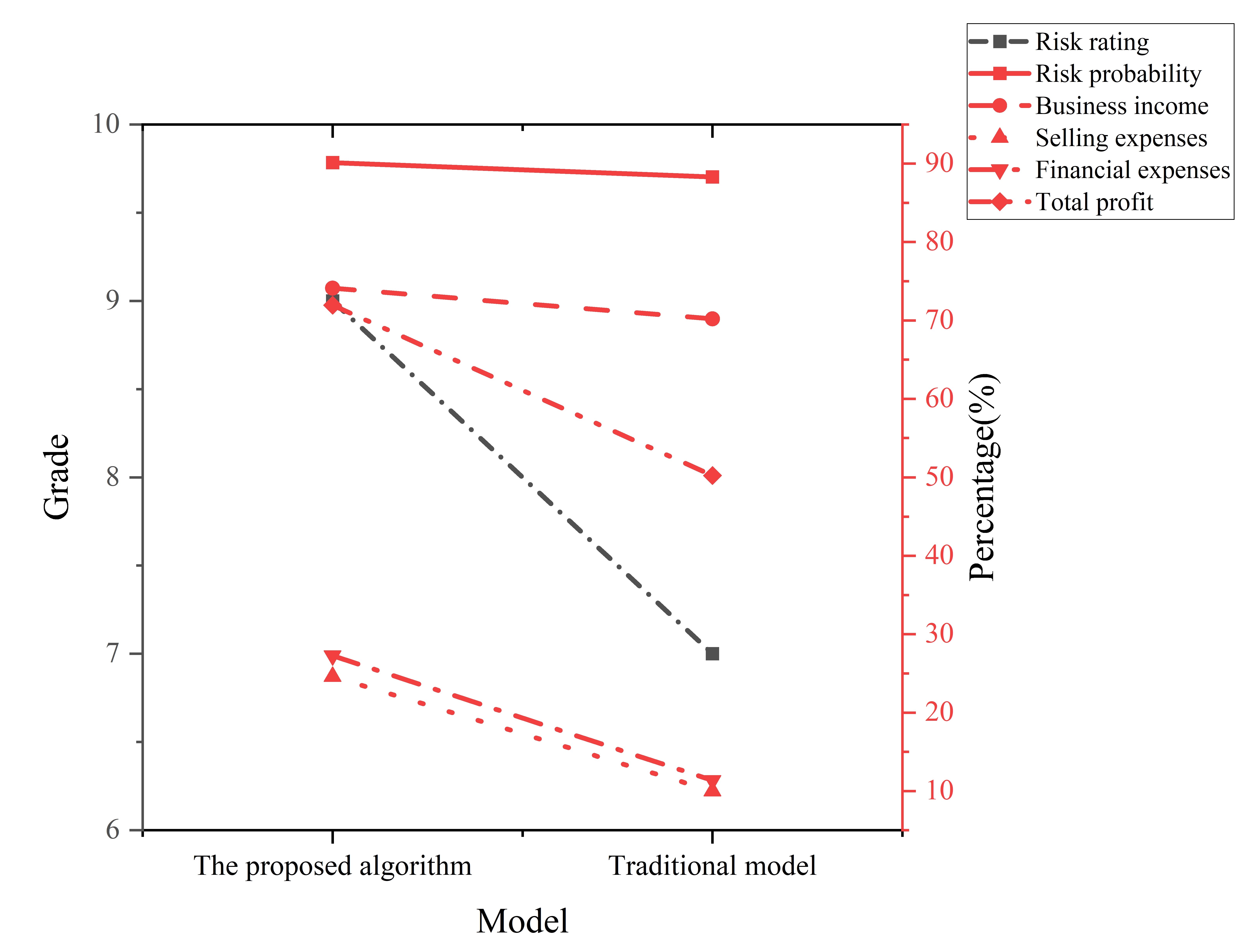

Supplement: S1 Data — (ZIP) [file pone.0300928.s001.zip › ╩2╛▌░n/Figure5.jpg]
